# Experience of danaparoid to treat vaccine-induced immune thrombocytopenia and thrombosis, VITT

**DOI:** 10.1186/s12959-021-00362-y

**Published:** 2022-02-04

**Authors:** Lasse Myllylahti, Hanna Pitkänen, Harry Magnani, Riitta Lassila

**Affiliations:** 1 Division of Internal Medicine and Rehabilitation, Department of Internal Medicine, Helsinki University Hospital, Helsinki, Finland; 2 Helsinki University, Division of Anesthesiology, Department of Anesthesiology , Intensive Care and Pain Medicine, University of Helsinki and Helsinki University Hospital, Helsinki, Finland; 3 Clinical Research Institute HUCH, Helsinki, Finland; 4 Independent Clinical Consultant, Schoutstraat, 54 Oss, The Netherlands; 5 Unit of Coagulation Disorders, Department of Hematology, Comprehensive Cancer Center, Helsinki University Hospital, and Research Program Unit in Systems Oncology, University of Helsinki, Haartmaninkatu 8, 00290 Helsinki, Finland; 6 Finnish Institute of Health and Welfare, Helsinki, Finland

**Keywords:** COVID19, Vaccination, PF4 antibody, VITT, Autoimmune HIT, Danaparoid sodium, Clinical case series

## Abstract

**Background:**

Vaccine-induced immune thrombocytopenia and thrombosis (VITT) is triggered by nCOV-19 adenovirus-vectored vaccines against SARS-CoV2. Pathogenesis has been mainly related to platelet activation via PF4-reactive antibodies that activate platelets and may cross-react with heparin. Data concerning optimal anticoagulation are anecdotal, and so far, there are scattered reports of danaparoid use in VITT management. Danaparoid has good efficacy and safety in treatment of heparin-induced thrombocytopenia. We report here our experience of the administration and monitoring danaparoid in VITT.

**Methods:**

We diagnosed a series of six hospitalized cases of VITT, based on the international diagnostic guidance. All VITT-related data were from the local electronic medical and laboratory record system and were analyzed with IBM SPSS Statistics.

**Results:**

Predominately women in their late 40’s developed VITT on average 24 days (range 9–59) after the first ChAdOx1 dose. Clinical presentation included single or multiple venous and/or arterial thrombosis, moderate thrombocytopenia and high D-dimer levels. After detecting PF4 antibodies subcutaneous danaparoid was our first-line antithrombotic treatment with an average duration of three weeks. The median plasma anti-FXa activity was in the lower part of the therapeutic range and during the first week of danaparoid administration clinical symptoms, platelet counts, and fibrin turnover resolved or significantly improved. The average duration of hospital admission was 10 days [2–18]. One patient died but the other five patients recovered completely.

**Conclusions:**

The clinical outcomes of our small cohort align with the earlier published reports, and support danaparoid as a rational option for the initial anticoagulation of VITT patients.

## Introduction

Global outbreak of severe acute respiratory syndrome coronavirus 2 (SARS-CoV-2) infections emerged early in 2020. The vaccination program has been critical in control of the pandemic due to its robust efficacy and safety [1–4]. However, in March 2021, concerns arose over emerging reports of immune thrombotic syndromes after nCOV19 adenoviral vector vaccination [5, 6]. Some patients suffered combined thrombocytopenia and a clinical course of multiple and/or unusually sited thrombosis, including cerebral venous sinus (CVST) and splanchnic vein thrombosis, as well as arterial events [5–7]. Most typical biomarkers included low platelet and high fibrin D-dimer levels and platelet-activating anti-PF4 antibodies (by ELISA method, rapid immunoassays are usually negative) without previous heparin exposure [7, 8]. Before the pandemic, anti-PF4 antibodies and thrombocytopenia were rare amongst patients suffering from CVST [9]. Clinical presentation mimicked the condition previously reported as autoimmune or spontaneous heparin-induced thrombocytopenia (aHIT) [10]. The condition is now known as vaccine induced immune thrombocytopenia and thrombosis (VITT) [ 8] or thrombosis with thrombocytopenia syndrome (TTS) by WHO [11].

Treatment options of VITT are based on the experience from other anti-heparin/PF4 antibody –related disorders of HIT and aHIT. Anticoagulation with preferably a non-heparin agent and administration of intravenous immune globulin (IVIG) are recommended to restrain the pathological platelet activation [8, 10]. The optimal anticoagulant for the initial administration is unclear, direct parenteral thrombin inhibitors, argatroban and bivalirudin, as well as danaparoid and fondaparinux are options, and the direct oral anticoagulants (DOACs) appear to be suitable, at least in the later course of the disease [8, 10, 11].

Danaparoid sodium is a non-heparin glycosaminoglycan antithrombotic that inhibits thrombin generation. It has been successfully used for HIT and its alternative administration routes (intravenous and subcutaneous) provide practical options for both inpatient and outpatient administration [12]. Unlike other agents, danaparoid is capable to detach PF4 from the platelet surface and disrupt PF4 containing immune complexes [10]. Hence theoretically, danaparoid should have a direct influence on VITT pathogenesis beyond its anticoagulant action [10, 13]. Our local guidance for initial anticoagulation during the acute treatment of HIT includes danaparoid, administered either intravenously (loading bolus 1250–3750 U with subsequent tapered infusion till 150–200 U / h) or subcutaneously at doses of 750–1500 U, 2–3 times a day with targeted anti-FXa activity of 0.3–0.5 U / mL [14]. There are a few reports of danaparoid use for treatment of HIT during COVID-19 [15, 16] or thrombosis post vaccination [17–20]. In this study, we want to share our experience of its use to treat VITT.

## Patients and methods

Our adapted diagnostic guidance requires previous nCOV19 adenovirus-vectored vaccination (usually 4–30 days before presentation), evidence of new thrombosis and thrombocytopenia and a positive anti-heparin/PF4 antibody ELISA test to confirm a diagnosis of VITT [8, 21].

Our study was accepted by the Helsinki University Ethical Committee (HUS/1238/2020). Written informed consents were received from patients 2 to 6 and from a close relative of patient 1. We collected all available VITT episode -related clinical and laboratory data from local electronic medical and laboratory record systems (EPIC Apotti, Weblab Clinical). IBM SPSS Statistics 25 was used to describe and analyze the collected data (Descriptive Statistics package) and Prism version 9 to visualize the data.

Our main focus was to evaluate all patients’ medical history, date of vaccination, prior heparin exposure (< 6 months before current presentation), initial clinical presentation with laboratory and coagulation biomarker statuses, initial antithrombotic medication and detection of anti-heparin/PF4 antibodies (ELISA, Asserachrom HPIA, Diagnostica Stago, France). In addition, administration of intravenous immune globulin (IVIG), clinical course during hospital admission, administration of danaparoid and its anti-FXa -activity (U/mL, HemosIL Liquis Anti-Xa, Mediq Suomi Oy), and final clinical outcome were recorded when examining the raw health information data. The aim of the anti-FXa –activity levels during subcutaneous danaparoid administration was 0.3–0.5 U/mL.

With respect to the systematic coagulation analysis, we screened coagulation times including prothrombin time (Medirox Owren’s PT (%) Medirox, Nyköping, Sweden), activated partial thromboplastin time (APTT (seconds, reference range 28–37 s) Actin FSL®, Siemens) and thrombin time (seconds, reference range 17–24 s BC Thrombin reagent, Siemens). Antithrombin activity (AT, (%) reference range 85–125%) was captured with a chromogenic assay (Berichrom Antithrombin III). We also analyzed fibrinogen level (g/L, reference range 2.0–4.0 g/L, Clauss method, HemosIL Q.F.A. Thrombin, Werfen, Barcelona, Spain), fibrin D-dimer level (mg/L, reference range < 0.5 mg/L, HemosIL D-Dimer HS 500, ILS Laboratories), coagulation factor VIII activity (FVIII:C, IU/dL, one-stage clotting assay, Pathromtin SL and FVIII Deficient Plasma)). Furthermore, D-dimer to fibrinogen ratio was calculated.

We collected available data at following 5 time points: the admission day (time point 1) and dynamically from days 1–3 from admission (time point 2), days 4–7 (time point 3), days 8–14 (time point 4) and days 15–30 (time point 5). These time points were matched with the dynamics of platelet count and an acute phase reactant C-reactive protein (CRP).

### Patient 1

A 40-year-old man with history of hypertension, obesity (145 Kg and BMI 40 Kg/m), type 2 diabetes, achalasia, and sleep apnea, was admitted to hospital 9 days after his first dose of ChAdOx1 with complaints of fever, arthralgia and chest pain. Thrombocytopenia (40 ×  10^9^/L), extreme fibrin turnover (D-dimer > 128 mg/L) and acute myocardial infarction (AMI) were identified. Contrast head computer tomography (CT) scan was diagnostic for CVST with extensive clot burden and secondary intracranial hemorrhage (ICH) due to increased venous pressure. After detection of anti-PF4 antibodies by ELISA, intravenous danaparoid (loading bolus of 1500 U with subsequent infusion of 250 to 330 U/h) was initiated and platelets, fresh frozen plasma and fibrinogen were supplemented because of continued bleeding. IVIG was administered and decompressive hemicraniectomy was performed. However, clinical course deteriorated, and the patient died two days after the admission.

### Patient 2

A 21-year-old woman with BMI of 30.3 Kg/m^2^ (94.5 Kg) was in remission from acute lymphoblastic leukemia (ALL) after allogenic stem cell transplantation. Beyond that, she had type 1 diabetes and chronic pain issues. She was admitted to hospital 12 days after her first ChAdOx1 dose with recurrent headache, elevated D-dimer (12 mg/L) and new thrombocytopenia (54 × 10^9^/L). Head MRI scan revealed extensive CVST. On day 5 after admission anti-PF4 antibodies were positive by ELISA with previous negative rapid test. Anticoagulation was switched from tinzaparin to subcutaneous danaparoid (1250 U × 2, later 1250 U + 750 U) and IVIG 0.4 g/Kg/day for five consecutive days was administered. This co-treatment led to recovery and the patient could be discharged after 15 days of admission with self-injections of danaparoid. Two months later anticoagulation was switched to fondaparinux for another month by which time the patient had fully recovered and a control head MRI scan was negative for residual CVST.

### Patient 3

A 52-year-old man with BMI of 24 Kg/m^2^ (77 Kg) with dyslipidemia, paroxysmal atrial fibrillation with low arrythmia burden (no prior anticoagulation) and aortic stenosis. 9 days after his first ChaAdOx1 dose he was admitted to hospital with headache and chest pain. AMI was diagnosed and percutaneous coronary intervention was performed. Thrombocytopenia (55 × 10^9^/L) and strongly elevated D-dimer (101 mg/L) were found. After detection of anti-PF4 antibodies by ELISA, IVIG 1 g/Kg/day for two consecutive days together with subcutaneous danaparoid (initially 750 U × 2, later 1250 U × 2) were initiated. During the early phase of admission, portal vein and cephalic vein thrombosis were diagnosed. The clinical course gradually ameliorated, and the patient was discharged 18 days after admission with ambulatory self-injected danaparoid and peroral ticagrelol. Later the patient was switched to apixaban and is still taking ticagrelol. He fully recovered.

### Patient 4

A 60-year-old woman had asthma and reflux esophagitis, and a history of bilateral pulmonary embolism (PE) 5 years earlier. Her BMI was 29.8 Kg/m^2^ (90 Kg). 19 days after her first ChAdOx1 dose she developed bilateral pulmonary emboli (PE) and a left tibial vein thrombosis. At this point, the platelet count was normal, and dalteparin anticoagulation was initiated. 41 days after vaccination she was re-admitted due to the onset of dizziness, headache, and nausea. CVST was excluded by contrast CT scan, but a new thrombocytopenia (54 × 10^9^/L) was detected. D-dimer was elevated (1.1 mg/L) and anti-PF4-antibodies were positive in ELISA. After danaparoid 1500 U × 2 sc was started the platelet count normalized and her clinical course improved without IVIG. The patient was discharged on day 7 after switching to oral dabigatran and indefinite anticoagulation was recommended because of the recurrent episode of PE. She fully recovered.

### Patient 5

A 68-year-old woman, with no significant medical history, complained, 16 days after the first ChAdOx1 dose, of recurrent headaches. Thrombocytopenia (65 × 10^9^/L) and markedly elevated D-dimer level (35 mg/L) were found. Head MRI scan showed an extended left side CVST, and she had also a small PE with minor symptoms. Danaparoid was initiated since anti-PF4 antibodies were positive by ELISA. IVIG was administered and the clinical course, platelet count and D-dimer level responded favorably. One week after admission, she was discharged with ambulatory subcutaneous self-injected fondaparinux. She made a full recovery, and later anticoagulation was switched to oral apixaban, the duration of which is still to be evaluated.

### Patient 6

A 42-year-old woman with sleep apnea and obesity (109 Kg with BMI 38 Kg/m^2^) was admitted to hospital 59 days after her first ChAdOx1 dose with recent symptoms of headache, common cold and myalgia. Initial laboratory evaluation identified thrombocytopenia (73 × 10^9^/L) and high D dimer (20.4 mg/L). Bilateral PE was diagnosed by contrast CT scan, and she was commenced on enoxaparin. The platelet count remained low, thrombo-inflammatory activity persisted, and her clinical course did not improve. Head MRI scan revealed a left internal jugular vein thrombosis and abdominal contrast CT scan identified extended portal vein thrombosis. Anti-PF4 antibodies were negative by rapid immunoassay but due to technical reasons initial ELISA samples were unavailable. However, clinical suspicion of probable late onset VITT was raised hence IVIG (1 g/Kg for two consecutive days) together with subcutaneous danaparoid were initiated. The thrombocytopenia and thrombotic activity recovered and her clinical course gradually improved. After two weeks of danaparoid anticoagulation she was commenced on oral apixaban for 12 months, and made a full recovery.

## Results

### Clinical data

In Finland, the ChAdOx1 nCOV-19 (Vaxzevria®, Astra Zeneca) has been used for all adenoviral COVID-19 vaccinations. By the end of the May 2021, the number of first ChAdOx1 doses was 358,000, and 55,000 people had completed their vaccination program with the two ChAdOx1 doses [22].

We diagnosed six VITT cases in Finland between mid-March and May 2021 after the first vaccination dose, and the use of this vaccine was discontinued in April 2021. According to international diagnostic criteria [8, 23], patients 1–3 and 5 were classified with definite and patients 4 and 6 with possible VITT, but we consider that all cases represent the spectrum of VITT disease. The mean age of our predominately female (4/6) patients was 47 years (range 21–68 years). The mean elapsed time from the first ChAdOx1 vaccination to the Emergency Department contact was 24 (range 9–59) days (Tables 1 and 2). Half of the patients had CVST (1, 2 and 3), but other sites of thrombosis were also verified (patients 1, 3, 4, 5 and 6). Arterial events were detected in two male patients, both cases being AMI; myocarditis as a differential diagnosis in patient 1. One patient had prior exposure to a heparin with her previous therapeutic dalteparin for a PE. The mean duration of hospital admission was 10 days (range 2–18 days). Five of the six patients fully recovered but one had a fatal outcome (patient 1).

**Table 1:** Baseline clinical characteristics and laboratory observations

Patient	1	2	3	4	5	6
Age	40	21	52	60	68	42
Sex	M	F	M	F	F	F
Admission – days after vaccination	9	12	9	41	16	59
Prior exposure to a heparin	No	No	No	Yes	No	No
Hemoglobin (M134–167, F 117–155 g/L)	147	106	146	116	N/A	N/A
WBC Count (× 10^9^/L) (3.4–8.2 × 10^9^/L)	6.1	5.5	5.4	5	N/A	N/A
Neutrophilia or monocytosis	Yes	No	Yes	Yes	N/A	N/A
ALT (U/L) (< 50 U/L)	127	12	55	55	N/A	N/A

(reference values), N/A = not available.

Nadir since admission to a distant hospital.

**Table 2:** VITT diagnosis, location of thrombosis, antithrombotic and IVIG therapy

Patient	*1*	2	3	4	5	6
Cerebral venous sinus thrombosis	Yes	Yes	No	No	Yes	No
Multiple thromboses	Yes	No	Yes	Yes	Yes	Yes
Arterial thrombosis	Yes	No	Yes	No	No	No
Anti-PF4 Ab ELISA positivity –Days after admission	1	5	1	0	1	N/A
Initial (1–2 doses) antithrombotic treatment	enoxaparin, aspirin	tinzaparin	enoxaparin, aspirin, ticagrelol	danaparoid	danaparoid	enoxaparin
IVIG	Yes	Yes	Yes	No	Yes	Yes
Duration of hospital stay (days)	2	15	18	7	7	20
Outcome	Fatal	Recovery	Recovery	Recovery	Recovery	Recovery

N/A = not available.

### Laboratory data

**Fig. 1:**
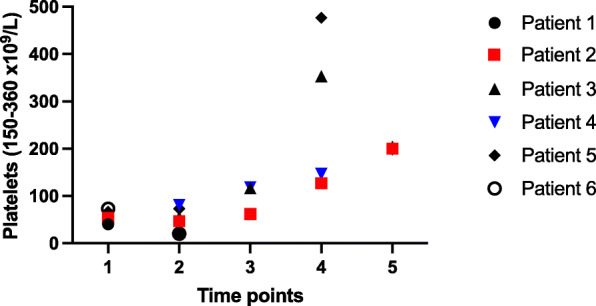
Evolution of platelet counts (normal range 150–360 × 10^9^ / L) before and during danaparoid therapy. Time points: 1 = on admission day, 2 = at 1–3 days, 3 = 4–7 days, 4 = 8–14 days, 5 = 15–30 days

**Fig. 2:**
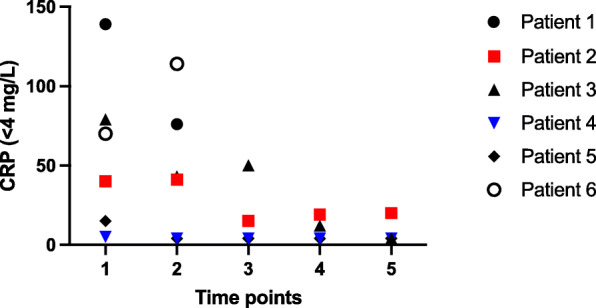
Evolution of C-reactive protein (normal < 4 mg/L) before and during danaparoid. Time points: 1 = on admission day, 2 = at days 1–3, 3 = days 4–7, 4 = days 8–14, 5 = days 15–30

The average hemoglobin level was 129 g/L (range 106–147 g/L) and all patients presented with moderate thrombocytopenia (mean count 57 × 10^9^/L, range 40–73 × 10^9^/L, Fig. 1) 7–14 days after admission platelet counts had normalized to an average count of 276 × 10^9^/L (range 127–477 × 10^9^/L). Available results of general biomarkers did not identify significant liver or renal impairment. White blood cell counts were normal in all cases, but the differential analysis showed neutrophilia or monocytosis in three patients. Almost every patient presented with inflammation based on the CRP levels (mean 55 mg/L; range 5–139 mg/L, Fig. 2). By timepoint 4–7 days from hospitalization, inflammation already significantly attenuated (mean CRP 19 mg/L; range 4–50 mg/L), excluding patient 6 whose diagnosis of VITT was delayed in another hospital.

**Fig. 3:**
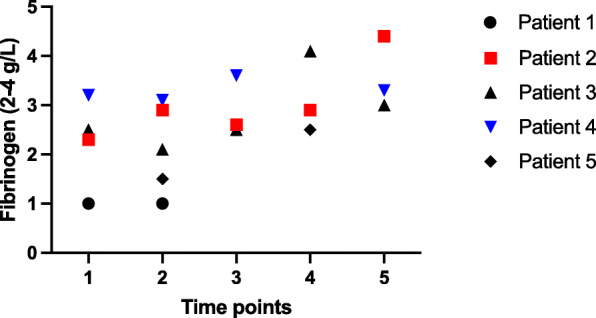
Fibrinogen (normal 2–4 g/L) levels before and during danaparoid therapy. Time points: 1 = on admission day, 2 = at 1–3 days, 3 = 4–7 days, 4 = 8–14 days, 5 = 15–30 days

**Fig. 4:**
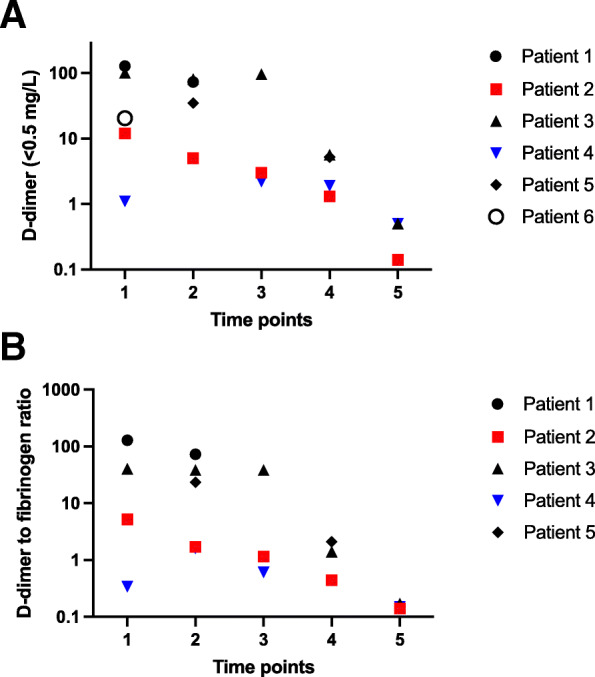
**A** and **B** Evolution of D dimer (normal < 0.5 mg/L) and D-dimer to fibrinogen ratio. Time points: 1 = on admission day, 2 = at 1–3 days, 3 = 4–7 days, 4 = 8–14 days, 5 = 15–30 days

We did not detect abnormalities in coagulation times of PT, APTT, thrombin time or antithrombin levels during and after the admissions. FVIII activity was elevated in all patients, peaking at 335 IU/dL in patient 3 at the time point 4. Similarly, compared with other reports of VITT coagulation abnormalities [7, 8], D-dimer levels were elevated in every patient, and values exceeding 30 mg/l, in patients 1,3 and 5, are compatible with extensive fibrin turnover ((see Fig. 4a). Furthermore, low fibrinogen levels identified in two patients (Fig. 3). D-dimer to fibrinogen ratio was extreme in patients 1 and 3, suggesting markedly enhanced fibrin degradation (Fig. 4b).

### Treatment data

**Fig. 5:**
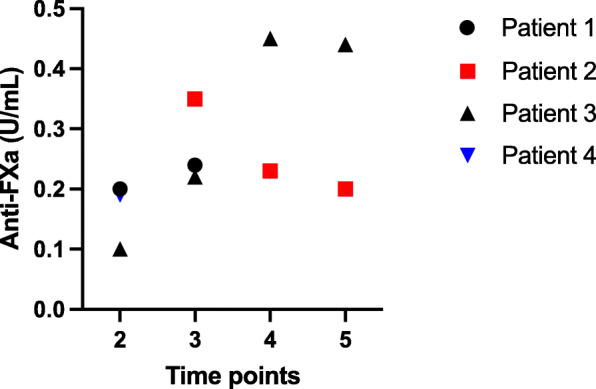
Available anti-FXa activity (U/mL) levels through the danaparoid treatment course. Time points: 1 = on admission day, 2 = at 1–3 days, 3 = 4–7 days, 4 = 8–14 days, 5 = 15–30 days

Five of the six patients were administered IVIG to reduce and prevent the further pathological platelet activation (Table 2). Danaparoid therapy and its follow up showed a favorable course (Table 3). Subcutaneous danaparoid was generally initiated at the early phase of the hospital admission with an average treatment duration of 20 days (range 1–60 days) with twice daily dosing. Average initial daily dose was 2333 U (range 1500–3000 U). Median anti-FXa activity levels remained at the lower range of recommended scale, 0.3 U/mL (Fig. 5). D-dimer levels after one week of danaparoid treatment significantly declined compared with the initial phase, and the one-week mean levels were 5.2 mg/L (range 1.7–11.8 mg/L). One clinically significant bleeding episode was associated with VITT as the CVST of patient 1 was complicated with progressive secondary ICH, already present before danaparoid initiation.

**Table 3:** Course and outcome of danaparoid treatment

Patient	1	2	3	4	5	6
Danaparoid initiation –Days after admission	1	6	2	0	0	4
Route of administration	iv	sc	sc	sc	sc	sc
Initial sc. daily dose (U)	No	2500	1500	3000	N/A	N/A
Anti-FXa (U/mL) Median	0.23	0.31	0.33	0.23	N/A	N/A
D-Dimer after 4–7 days of danaparoid therapy	N/A	1.7	11.8	1.9	5.2	N/A
Bleeding events	Yes*	No	No	No	No	No
Outcome	Fatal	Recovery	Recovery	Recovery	Recovery	Recovery

* = ICH, N/A = not available

## Discussion

Our Finnish VITT cohort seems to be aligned with the earlier reports with respect to: clinical presentation, coagulation biomarker status and clinical outcome. Our mortality rate is close to the rate recently reported for a large UK cohort [23] (17 vs 22%), and the only fatal outcome was the very first VITT case of the nation. At that time, an optimal treatment protocol, including upfront IVIG as the key to pathogenesis control, was only developing. In patients with CVST-VITT, higher mortality rates (22–61%) are reported compared with VITT in general [23–26]. The potential of VITT for devastating outcomes calls for rapid recognition of cases. Since recognition of this new syndrome, several interim guidelines have been published to help frontline health workers and clinicians [8, 11, 21, 27]. As a result improved identification of VITT and its management have led to a reduction in mortality [28].

Danaparoid, most frequently administered subcutaneously in our cohort, is a reasonable option for initial VITT anticoagulation supported by previously published guidance and experience from other anti-H/PF4 antibody – related disorders [8, 10–12]. Due to its low overall negative charge density compared with heparin the ultra-large complexes with PF4 and the platelet activating antibodies will not form unlike in HIT. In addition, the absence of heparin-like domains explains its low propensity to cross-react with anti-PF4/heparin antibodies.

Our small study suggests that danaparoid administration and upfront IVIG were effective for VITT treatment since five of the six patients fully recovered without significant clinical sequelae. Subcutaneous administration is more practical to handle than continuous intravenous infusions, which direct thrombin inhibitor anticoagulants require. The effective subcutaneous treatment is easy to extend to the ambulatory mode, if needed. However, intravenous danaparoid, even at low infusion rates provides constant antithrombotic and anti-inflammatory activity levels compared with the peaks and troughs of intermittent subcutaneous injections and is the preferred option in severe cases. Our patients´ danaparoid was monitored with anti-FXa activities, which were in the lower level of the target therapeutic range. The only bleeding complication was progression of an ICH which had occurred before danaparoid treatment initiation. This patient’s high intravenous infusion rate coupled with a loading bolus of danaparoid was probably too intensive. Intracranial bleeding during CVST has previously proved to be a significant risk factor for detrimental outcome [29], also reported in the largest available VITT cohort [23]. Our results also compare favorably with previously reported use of danaparoid to treat VITT [17–20].

Our study has certain limitations. The sample size is only six patients and data are retrospective and observational with some missing data points (especially patients 5 and 6). Upon VITT diagnosis all patients received danaparoid so there is no comparison with other initial anticoagulant options. The availability of danaparoid is a national and tradition –based policy reserved for patients, who are intolerant or allergic to heparin.

We did not use direct oral anticoagulants (DOAC) early on, as multiple, and also arterial thrombi had to be managed. In addition, despite some favoring data [30, 31], there is scanty evidence concerning the safety and efficacy of DOACs in the management of these forms of thrombosis (i.e, CVST). Dabigatran has proven at least as good option as warfarin [31], which is not recommended in acute HIT due to its impairment of protein C and S [10, 32].

To establish the optimal first-line anticoagulation of VITT and other PF4 antibody –related disorders, more research is needed, including prospective comparative studies and pathophysiological research [33]. Even if the role of adenoviral vector nCOV19 vaccination has diminished in many countries, it is important to gain and publish knowledge of VITT for future occasions. Although we recognize the pathogenetic aspects of VITT, we do not understand who will get it, and the syndrome is not limited to adenovirus vector nCOV19 vaccine exposure only. Rare but potentially disastrous immune thrombotic anti-H/PF4 antibody related syndromes are also likely to occur in clinical scenarios that do not involve vaccination [6].

## Conclusions

Our clinical case series suggests that danaparoid may be a rational option for initial anticoagulation of VITT together with upfront IVIG. Danaparoid has pharmacodynamic advantages and seemed to be well tolerated by our patients.

## Data Availability

We are open for data sharing, please contact the corresponding author (riitta.lassila@hus.fi) if needed.
